# Effects of Soil Water Deficit on Insecticidal Protein Expression in Boll Shells of Transgenic Bt Cotton and the Mechanism

**DOI:** 10.3389/fpls.2017.02107

**Published:** 2017-12-11

**Authors:** Xiang Zhang, Jian Wang, Sheng Peng, Yuan Li, Xiaofeng Tian, Guangcheng Wang, Zhongning Zhang, Zhaodi Dong, Yuan Chen, Dehua Chen

**Affiliations:** Jiangsu Provincial Key Laboratory of Crop Genetics and Physiology, Yangzhou University, Yangzhou, China

**Keywords:** Bt cotton, soil water deficit, gene expression, insecticidal protein, nitrogen metabolism

## Abstract

This study was conducted to investigate the effects of soil water deficit on insecticidal protein expression in boll shells of cotton transgenic for a Bt gene. In 2014, Bt cotton cultivars Sikang 1 (a conventional cultivar) and Sikang 3 (a hybrid cultivar) were planted in pots and five soil water content treatments were imposed at peak boll stage: 15% (G1), 35% (G2), 40% (G3), 60% (G4), and 75% field capacity (CK), respectively. Four treatments (G2, G3, G4, and CK) were repeated in 2015 in the field. Results showed that the insecticidal protein content of boll shells decreased with increasing water deficit. Compared with CK, boll shell insecticidal protein content decreased significantly when soil water content was below 60% of maximum water holding capacity for Sikang 1 and Sikang 3. However, increased Bt gene expression was observed when boll shell insecticidal protein content was significantly reduced. Activity assays of key enzymes in nitrogen metabolism showed that boll shell protease and peptidase increased but nitrogen reductase and glutamic-pyruvic transaminase (GPT) decreased. Insecticidal protein content exhibited significant positive correlation with nitrogen reductase and GPT activities; and significant negative correlation with protease and peptidase activities. These findings suggest that the decrease of insecticidal protein content associated with increasing water deficit was a net result of decreased synthesis and increased decomposition.

## Introduction

Although Bollgard II^®^ varieties containing the additional δ-endotoxin of *Bacillus thuringiensis*, Cry2A (b), have been employed in both Australia and the United States (Morse, 2016), the single toxin Bt-cotton (Bollgard I) has dominated domestic production in Asia and Africa (Huang et al., 2010; Clive, 2012). People still use the single toxin Bt-cotton, expressing its own insecticidal protein, to effectively reduce losses to pests such as cotton boll worm (Sujii et al., 2013), and to reduce pesticide use in those countries. The use of Bt-cotton produces dramatic economic and ecological benefits (Dhillon and Sharma, 2013). Therefore, it is vital to ensure the expression of the insecticidal protein in Bt cotton.

Research has suggested that Bt cotton insecticidal protein is not expressed steadily (Knox et al., 2006; Addison and Rogers, 2010; Hallikeri et al., 2011; Wang D. M. et al., 2012; Kumar et al., 2013). Soil water deficit significantly affects insecticidal protein expression in the leaves of Bt cotton (Rochester, 2006; Parimala and Muthuchelian, 2010). Increased damage to Bt cotton by cotton worm in Shandong and Hebei provinces of China in 2005 and 2006 may have been due to lack of rain and a resulting soil water deficit from June to July (Liu et al., 2008). Likewise, Carter et al. (1997) and Benedict et al. (1996) found that lack of rain resulted in soil water deficit and associated water stress reduced the content of total soluble protein and insecticidal protein in June and July. Drought stress could lead to DNA degradation in cotton seedling tissues, producing many residual DNA fragments that could inhibit the synthesis of functional proteins and structural proteins (Yang et al., 2016). Thus, several lines of independent evidence implicate drought stress in the failure of insect resistance of Bt cotton. In most of the world, drought is an important problem during the cotton whole growing period (Li et al., 2010).

Environment may influence insect resistance of Bt cotton in a number of ways. One hypothesis suggested that under an adverse environment, DNA methylation of the promoter regions of the Bt gene switches off gene expression (Stam et al., 1997). Another hypothesis suggested that tannin, generated by cotton plants exposed to adverse environments, was binding to Bt insecticidal protein and inactivating it (Holt, 1998). A third hypothesis suggested the protein synthesis decreased, resulting in decreased Bt insecticidal protein content (Chen et al., 2004, 2005). They found there was a significant positive linear correlation between GPT activity and insecticidal protein, while a significant negative linear correlation between peptidases activity and insecticidal. However, we do not know how soil water deficit affects the expression of Bt insecticidal protein in bolls, and what mechanism is responsible for these effects.

The present work was undertaken to study the effects of soil water deficit on insecticidal protein expression in boll shells of transgenic Bt cotton, enzyme activities in nitrogen metabolism, and consequences of altered enzyme activities for expression of the Bt gene. The resulting information is expected to provide a scientific basis for improving control of cotton boll worm under adverse circumstances.

## Materials and Methods

### Test Materials and Design

The experiment was carried out in the Key Laboratory of Genetics and Physiology of Jiangsu Province, Yangzhou University, China (32°30′N, 119°25′E) in 2014 and 2015. Two Bt transgenic cotton (*Gossypium hirsutum L*) cultivars medium in maturity, Sikang 1 (SK1), a conventional Bt cultivar; and Sikang 3 (SK3), a hybrid Bt cultivar, were used in this study.

Seeds of the two cultivars were planted on April 7th in a warm room covered by plastic film. In 2014, on May 20th the seedlings were transplanted to porcelain pots (50-cm height, 40-cm diameter, 62.8-L volume) filled with 20 kg sandy loam soil [Typic fluvaquents, Entisols (U.S. taxonomy)] obtained from the field and containing 18.5 g kg^-1^ organic matter and 108, 40.5, and 82.0 mg kg^-1^ available N-P-K respectively. On the day of transplanting, 1.6 g N as urea, 0.6 g P as single superphosphate and 2.4 g KCl were mixed into the soil of each pot, and one seedling was transplanted to each pot. At 50 days after transplanting, 1.54 g N as urea, 0.6 g P as single superphosphate and 2.4g K as KCl were top-dressed into each pot. At 72 days after transplanting, 1.9g N as urea was top-dressed into each pot. Each variety was transplanted to 40 pots respectively.

In 2015, seedlings were transplanted to the field on May 20th at a row spacing of 0.85 by 0.33 m. The plot area was 32 m^2^. The field soil was the same as 2014. N (60 kg ha^-1^ as urea), P (300 kg ha^-1^ as single superphosphate), and K (120 kg ha^-1^ as KCl) were applied before transplanting. N (54 kg ha^-1^ as urea), P (300 kg ha^-1^ as single superphosphate), and K (120 kg ha^-1^ as KCl) were also applied at early flowering. Nitrogen as urea was also applied at the early boll development stage (126 kg ha^-1^ as urea) and at peak boll stage (30 kg ha^-1^). A split plot test design with three replications was used in the field. Fertilization regimes used in both years followed local recommendations.

### Preparation of Samples

In 2014, we set up 5 soil water treatments at the bolling stage: 15% (G1), 35% (G2), 40% (G3), 60% (G4) and 75% field capacity (CK) respectively. In 2015, we repeated G2, G3, G4, and CK treatments, each with three repetitions.

We started to control watering 10 days before flowering – if it rained, the pots were moved into a room in 2014 and the field was covered by a rain-shelter in 2015. In both years, soil moisture content was monitored by HH2 Moisture meters (WET2, Delta-T Devices, Ltd., United Kingdom). If the meter found that the soil moisture content was lower than the designated value, we watered the soil in the morning, noon, and evening.

Cotton plants were selected for the experiment on July 20, 2014 and July 22, 2015, marking the inner surrounding 1–2 fruit nodes, and carrying on the water treatment for 10 days after anthesis. After 4 days of stress, the marked bolls were harvested, frozen by liquid nitrogen, and put into the -72°C ultra-low temperature freezer to save for assays.

### Determination of Bt Protein Content

The Cry1Ac protein concentrations in cotton boll shell extracts were determined by immunological analysis (ELISA) (Chen et al., 1997).

Three subsamples of boll shell (0.5 g FW) were prepared by homogenizing the frozen tissue in 2 ml extraction buffer (Na_2_CO_3_ 1.33 g, DTT 0.192 g, NaCl 1.461 g, Vitamin C 0.5 g dissolved in 250 ml distilled water) in 5 ml centrifuged tube. The contents of this tube were shaken with hand, and stored at 4°C for 4 h. The supernatant was collected after centrifugation at 11,180 × *g* at 4°C for 20 min, passed through a C18 Sep-Pak Cartridge (Waters, Milford, MA, United States), and 50 μL of subsample was used to estimate Cry1Ac contents. Quantification of Cry1Ac levels in combined samples was conducted using a commercially available Kit (Scientific Service, Inc., China Agriculture University, China). Microtitration plates were coated with the standard Cry1Ac insecticidal proteins and samples, and incubated at 37°C for 4 h. The antibodies were added to each well and incubated for a further 30 min at 37°C. The antibodies against the Cry1Ac insecticidal protein were obtained as described by Weiler et al. (1981). Horseradish peroxidase-labeled goat antirabbit immunoglubolin was added to each well and incubated for 30 min at 37°C. Finally, the buffered enzyme substrate (1,2-Phenylenediamine) was added and the enzyme reaction was carried out in the dark at 37°C for 15 min, and then terminated using 3 mol L^-1^ H_2_SO_4_. The absorbance was recorded at 490 nm. Calculation of Cry1Ac protein concentrations from the ELISA data was performed as described by Weiler et al. (1981). The other chemicals were supplied by Scientific Service, Inc., China Agriculture University, China.

### Real Time Fluorescence Quantitative PCR

#### Extraction and Purification of Total RNA from Plants

Extracting and purifying the total RNA of plants used the plant RNA extraction test kit [DP432, Tiangen Biotech (Beijing) Co., Ltd., China], following the manufacturer’s instructions.

#### Inverse Transcription

Using quantitative cDNA first chain synthesis kit [CWBIO, Tiangen Biotech (Beijing) Co., Ltd., China], and 5 μl RNA samples, 2.5 μL units oligo DT (L) and 9.625 μL RNAse-free ddH_2_0 were added in the 0.2 ml PCR thin-walled tube without the RNA enzyme, denatured 5 min at 70°C Celsius, then quickly placed on ice to cool for 5 min, in order to join the reverse transcription just as listed by **Table 1**, system for 25 L (the other reaction systems also were done according to this proportion) in the PCR instrument for 42°C reaction for 60 min.

**Table 1:** Components of the reverse transcription system and their volumes.

Compositions	Volume (μL)
5 × MLV RT reaction buffer	5
dNTPs	1.25
RRI (RNase inhibitor)	0.625
MLV reverse transcriptase	1

#### Real Time Fluorescence Quantitative PCR

Using SYBR GREEN I (Molecular Probes) [Takara Biotech (Beijing) Co., Ltd., China] of Bio as a fluorescence probe, real-time fluorescence quantitative PCR was performed in an ABI7500 real-time PCR system according to the manufacturer’s instructions. The primer sequences for the Bt gene are Bt-F: 5′-GTTCTGCCCAAGGTATCGAA-3′ and Bt-R:5′-GCAACGATACGTTGTTGTGG-3′. The qRT-PCR reactions were 15 μL, including 7.5 μL SYBR^®^Premix Ex TaqTm (2x) [AidLab Biotechnologies Co., Ltd., China], 0.3 μL forward primer (10 μm), 0.3 μL reverse primers (10 μm), 0.3 μL Rox reference dye II (50×), 1.2 μL cDNA and 5.4 μL ddH_2_O. In the fluorescence quantitative PCR instrument in accordance with the two step amplification process, thermal profiles of reactions were 95°C 30 s; 95°C 5 s, 60°C 35 s, 40 cycles. At last, the dissolution curve of real time PCR was determined. Each sample was repeated three times. According to the Ct value of the specific fluorescence threshold of each sample, the relative expression levels of different products were analyzed by the computing method of 2^-Δ ΔC_t_^.

### Assay of Enzyme Activities

#### Nitrate Reductase (NR) Activity

Nitrate reductase activities in boll shells or seeds were determined by the method of Nicholas and Deering (1976). Boll shells (0.2 g) were vacuum infiltrated in 10 mL incubation buffer of pH 7.5, 0.1 mol L^-1^ potassium phosphate, 0.05 mol L^-1^ KNO_3_, 1% [v v^-1^] propanol. The mixture was incubated in a shaking water bath at 30°C for 30 min in the dark. Then, 0.4 mL of the incubation buffer was diluted with water to 4 mL, and 1% sulfanilic acid in 1.5 mol L^-1^ HCl (2 mL) and 200 mg L^-1^ *N*-(1-naphthyl) ethylenediamine-HCl (2 mL) were added to stop the enzyme reaction. The mixture was then incubated for at least 20 min at room temperature to allow full color development. The absorbance was recorded at 540 nm.

#### Glutamic-Pyruvic Transaminase (GPT) Activity

The boll shell samples (1.0 g) were homogenized in 5 ml 0.05 m mol L^-1^ Tris-HCl pH 7.2, and the mixture was centrifuged at 26,100 × *g* for 10 min at 4°C. The supernatant solution was the crude extract for GPT. First, 0.2 mL of the supernatant was added to a mixture containing 0.5 mL of 0.8 mol L^-1^ alanine in 0.1 mol L^-1^ Tris-HCl (pH 7.5), 0.1 mL of 2 m mol L^-1^ pyriodoxal phosphate solution, and 0.2 mL of 0.1 mol L^-1^ 2-oxoglutarate solution. The reaction mixture was incubated at 37°C for 10 min., and 0.1 mL of a 0.2 mol L^-1^ of trichloroacetic acid solution was added to stop the reaction. Then, the pyruvate was converted to pyruvate hydrazine with chromogen. The color intensity of the hydrazine in saturated water toluene was read at 520 nm. GPT activity, in terms of pyruvate production, was calculated from authentic pyruvate standards run simultaneously (Thomas, 1975).

#### Protease and Peptidase Activities

The boll shell samples (0.8 g) were homogenized at 4°C in 1 mL of β-mercaptoethanol extraction buffer, (a mixture of ethylene glycol, sucrose, and phenylmethyl sulfonyl fluoride, pH = 6.8). Cell debris was removed by centrifugation, and the supernatant was placed on ice and immediately used to estimate protease activity. Protease activity was determined spectrophotometrically at 400 nm using azocasein as a substrate (Vance et al., 1979) and expressed in mg protein g^-1^ fresh weight (FW) h^-1^. Peptidase activity was determined as described by Wei et al. (2016). A total of 0.1 mL extract was added to 1 mL buffer containing 50 mM Tris-HCl (pH 8.0), 1 m Mol L^-1^ MnCl_2_ and 5 m Mol L^-1^ peptide, then 25 mL reaction mixture was incubated at 37°C for 30 min in 1 mL of a 1% ninhydrin solution containing 100 mg of cadmium acetate, 85 mL of ethanol, and 15 mL of acetic acid in a total volume of 100 ml. The optical density was measured at 505 nm.

Except as otherwise noted, chemicals were all supplied by Sinopharm Group Co., Ltd., China.

### Statistical Analysis

The data were processed and plotted with Excel 2003 software, and analyzed by SPSS 13.0 software package. All experiments were performed in triplicate, using two-way ANOVA with multiple comparisons to discern statistically significant differences at *p* < 0.05.

## Results

### Effect of Water Deficit on the Content of Insecticidal Protein in Cotton Boll Shells

**FIGURE 1:**
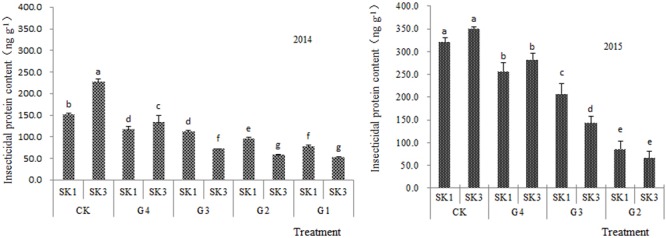
Effects of water deficit on the content of insecticidal protein in boll shells. Different lower-case letters indicate significant differences at the 0.05 level among treatments.

Soil water deficit significantly affected the contents of insecticidal protein in the boll shells of two Bt cotton varieties (**Figure 1**). Compared with the control, the contents of insecticidal protein in Bt cotton boll shells were decreased under water deficit treatment in both 2014 and 2015. The content of insecticidal protein in the boll shell gradually decreased with increased intensity of water deficit. Specifically, compared with the control, the contents of insecticidal protein at G4, G3, G2, and G1 decreased in SK1 by 22.5, 26.3, 36.1, and 48.8%; and in SK3 by 41.6, 68.6, 74.7, and 76.7%, respectively.

The reduction of insecticidal protein was larger for SK3 than SK1 under water deficit stress. These results were virtually identical for the 2 years of the study. Water deficit treatments of SK3 showed an average decrease of 65.4% comparing with the control, distinctly larger than the 33.4% of SK1. Under G3, the content of insecticidal protein in the cotton boll shell in both years was distinctly higher in SK1 than SK3.

Analysis of variance (**Table 2**) showed that the differences among insecticidal protein levels at different water deficit treatments were statistically significant, the insecticidal protein content of G4 being significantly lower than that of CK.

**Table 2:** ANOVA and Duncan’s test of the content of insecticidal protein in cotton boll shells.

Water	Insecticidal protein content		Cultivars	Insecticidal protein content	
deficit	(ng⋅g^-1^FW)			(ng⋅g^-1^FW)	
	2014	2015		2014	2015
CK	188.97a	335.75a	SK1	110.47a	217.63a
G4	124.79b	269.11b	SK3	108.31a	210.12a
G3	91.27c	174.79c			
G2	76.89d	75.85d			
G1	65.04e	–			
*F*-value	520.77	143.41	*F*-value	0.59	4.87

*G1, G2, G3, G4, CK represent 15, 35, 40, 60, and 75% field capacity, respectively. Different small letters in a column indicate significant difference at *P* < 0.05.*

In summary, soil water content at 60% of the maximum water holding capacity was a sufficient reduction that the insecticidal protein content in Bt cotton boll shells decreased significantly. SK1 showed a smaller decline than SK3.

### Effect of Water Deficit on the Expression of a Bt Gene in Cotton Boll Shells

**FIGURE 2:**
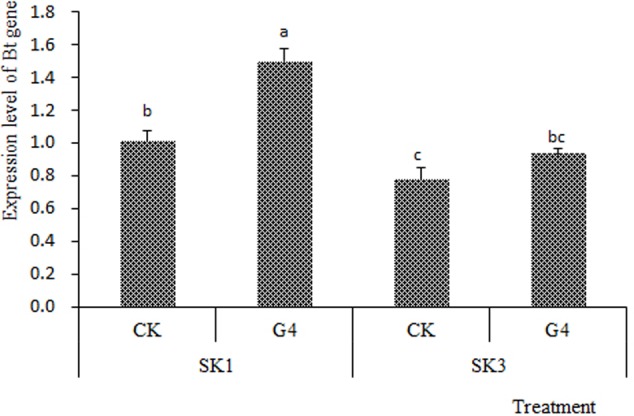
Effects of 96 h under water deficit on expression level of a Bt gene in cotton boll shells.

Further analysis (**Figure 2**) showed that after 96 h under G4 stress, Bt gene expression levels in boll shells of SK1 and SK3 were significantly increased. Compared with the respective controls, Bt gene expression levels of SK1 and SK3 increased by 48.6 and 22.1%, respectively. The increase of Bt gene expression was more for SK1 (a conventional cultivar) than SK3 (a hybrid cultivar).

Analysis of variance (**Table 3**) showed that, compared with the control (CK), the 40% Bt gene expression increase in the boll shell for G4 was statistically significant. The expression increase for the conventional cultivar (SK1) was also significantly higher than that for the hybrid cultivar (SK3).

**Table 3:** ANOVA and Duncan’s test of the expression level of a Bt gene in cotton boll shells (2015).

Water	Expression level	Cultivars	Expression
deficit	of Bt gene		level
CK	0.89b	SK1	1.25a
G4	1.21a	SK3	0.85b
*F*-value	46.62	*F*-value	72.58

*G4, CK represent 60% and 75% field capacity respectively. Different small letters in a column indicate significant difference at *P* < 0.05.*

### Physiological Characteristics of Nitrogen Metabolism

#### Activities of NR and GPT

The activities of NR and GPT enzymes in boll shells of SK1 and SK3 showed similar trends to one another. Compared with the respective controls, NR and GPT activities in boll shells decreased under soil water deficit (G4) by 19.1% and 17.2%, respectively for SK1, and by 24.8 and 15.6% for SK3 (**Table 4**). Analysis of variance (**Table 4**) showed that the activities of these synthetases related to nitrogen metabolism in boll shells declined significantly after Bt cotton was exposed for 96 h to soil water deficits of several intensities. Different cultivars had no significant difference of NR activity, but the activity of GPT was significantly higher for SK3 than SK1.

**Table 4:** Effects of water deficit on activities of nitrate reductase and glutamic-pyruvic transaminase in cotton boll shells (2015).

Cultivars		Treatment	NR activities	GPT
			(μmol⋅g^-1^⋅h^-1^)	activities
				(μmol⋅g^-1^⋅h^-1^)
SK1		CK	3.82a	15.1a
		G4	3.09a	12.5c
		G3	2.61a	8.6d
		G2	1.33c	6.2e
SK3		CK	4.04a	18.0a
		G4	3.04a	15.2a
		G3	2.88a	12.6c
		G2	1.32c	8.7d
ANOVA	Water deficit	CK	3.93a	16.6a
		G4	3.07a	13.9a
		G3	2.75a	10.6c
		G2	1.33c	7.5d
		*F*-value	64.5	40.3
	Cultivars	SK1	2.71a	10.6a
		SK3	2.82a	13.6a
		*F*-value	1.6	7.2

*G1, G2, G3, G4, CK represent 15, 35, 40, 60, and 75% field capacity, respectively. Different small letters in a column indicate significant difference at *P* < 0.05.*

#### Activities of Peptidase and Protease

The trends of changes in peptidase and protease activities were opposite to that of Bt protein content (**Table 5**). The activities of peptidase and protease in the boll shell for SK1 and SK3 both increased significantly under soil water deficit (G4). The percentage increase of SK3 was higher than that of SK1. Compared with the respective CK, the activities of peptidase and protease increased by 39.7 and 65.2% respectively in SK1, and by 41.9 and 92.9% respectively in SK3.

**Table 5:** Effect of water deficit on peptidase and protease activities in cotton boll shells.

Cultivars	Treatment		Peptidase	Protease
			activities	activities
			(μmol⋅g^-1^⋅h^-1^)	(μg⋅g^-1^⋅h^-1^)
SK1		CK	1.41e	33.74f
		G4	1.97d	55.74e
		G3	2.15c	70.12d
		G2	3.45a	104.85b
SK3		CK	1.60e	43.68e
		G4	2.27c	84.25c
		G3	2.81b	106.59b
		G2	3.48a	143.12a
ANOVA	Water deficit	CK	1.51c	38.7d
		G4	2.12b	70.0c
		G3	2.48b	88.4b
		G2	3.47a	124.0a
		*F*-value	13.8	24.4
	Cultivars	SK1	2.25b	66.1b
		SK3	2.54a	94.4a
		*F*-value	19.6	42.3

*G1, G2, G3, G4, CK represent 15, 35, 40, 60, and 75% field capacity, respectively. Different small letters in a column indicate significant difference at *P* < 0.05.*

Soil water deficit (G4) significantly reduced the activities of peptidase and protease, enzymes that function in protein decomposition, of Bt cotton in boll shells. The two enzyme activities were significantly higher in SK3 than SK1 boll shells. These results indicate that under soil water deficit, the degradation rate of boll shell protein increased, and was significantly higher for SK3 than SK1.

### Relationship between Insecticidal Protein Content and Nitrogen Metabolism

Insecticidal protein content in boll shells showed significant positive correlation with NR and GPT activity, and significant negative correlation with peptidase and protease activity (**Table 6**). These results further indicated that under soil water deficit stress, protein synthesis decreased and protein degradation increased, in total conferring a decrease of insecticidal protein concentration.

**Table 6:** Correlation coefficients between insecticidal protein content and key enzyme activities in cotton boll shells under water deficit stress.

Cultivars	Nitrate reductase activities	GPT activities	Peptidase activities	Protease activities
SK1	0.999^∗∗^	0.963^∗∗^	–0.990^∗∗^	–0.999^∗∗^
SK3	0.915^∗^	0.981^∗∗^	–0.984^∗∗^	–0.971^∗∗^

*^∗∗^ and ^∗^ represent significance at the 1 and 5% level, respectively.*

## Discussion

Previous studies have shown that soil water deficit reduced the insecticidal protein content of Bt cotton. Wang et al. (2001) and Blaise and Kranthi (2011) found that soil water deficit decreased insecticidal protein content in leaves and young squares, but the effect on the boll has not been reported. However, Martins et al. (2008) found that the insecticidal properties in the leaf and square had not changed significantly when soil water content was from 25 to 30% of maximum water holding capacity. By imposing different degrees of soil water deficit stress for 96 h, we found that soil water deficit resulted in decreased insecticidal protein content in boll shells of cultivars SK1 and SK3. The extent of decline in insecticidal protein content increased with the intensity of soil water deficit. When the two cultivars were subjected to soil moisture content of 60% of maximum water holding capacity, insecticidal protein contents were significantly lower than the respective controls (75%). Thus, the present data indicate that reduction of soil moisture content from 75 to 60% of maximum water holding capacity crosses a threshold of soil moisture, resulting in decreased boll insecticidal protein content of Bt cotton. Therefore, in the production of Bt cotton, soil moisture of lower than 60% of maximum water holding capacity in the cotton bolling period may reduce effectiveness of insect control.

Different Bt cotton cultivars respond to soil water deficit to different degrees. The decline of insecticidal protein content in boll shells of SK1 was lower than that of SK3 under soil water deficit. In normal water conditions (soil water content at 75% of maximum water holding capacity), the content of insecticidal protein in the boll shell was significantly higher in SK3 than SK1. When soil moisture content dropped to 45% or less of maximum water holding capacity, the content of insecticidal protein in the boll shell was significantly higher in SK1 than SK3. These results indicated that the stability of insecticidal protein content in reproductive organs was higher in SK1 than SK3. That was supported by Dong and Li (2007). Thus, SK1, which had higher insect resistance during drought, would be the preferred variety in dry years.

The mechanism of decline of Bt cotton resistance under adverse environments has not yet been established, but three hypotheses have been suggested: one proposes that the Bt gene promoter experiences methylation inactivation, which makes Bt expression switch off (Stam et al., 1997); a second suggests that the insecticidal protein combines with tannin, produced by the cotton plant under adverse conditions, losing activity (Holt, 1998). The third hypothesis suggests that decreased protein synthesis resulted in reduced expression of insecticidal protein under adversity (Chen et al., 2005).

The present results provide strong evidence against the hypothesis that the Bt gene promoter experiences methylation inactivation, which makes Bt expression switch off (Stam et al., 1997). Indeed, after 96 h under G4 stress, Bt gene expression levels in boll shells of SK1 and SK3 increased significantly (**Figure 2**), although the contents of Bt insecticidal protein decreased significantly.

The present results also showed that transcription and translation of the Bt gene had independent and complex regulation, and their correlation was low (Washburn et al., 2003; Hudson and Edwards, 2016). As the main driver of life activities, study at the protein level could provide more direct and accurate messages than those at the gene expression level (Zhu et al., 2003). Under drought conditions, we showed insecticidal protein content to decline less for SK1 than SK3, while Bt gene expression level increased more for SK1 than SK3. Although mRNA transcription increased under the adverse environment, translation efficiency might have decreased or protein degradation accelerated, causing a net decrease of insecticidal protein content. This is supported by our findings that the activities of peptidase and protease enzymes increased under stress. The increase of Bt expression level in boll shells might be a direct response to soil water deficit. The greater increase for SK1 than SK3 may reflect greater adaptability of SK1 to soil water deficit, and mitigate the decline of insecticidal protein content in boll shells.

From the view of nitrogen metabolism, previous studies have found that the decline of Bt-insecticidal protein content in leaves was related to reduced protein synthesis ability and increased decomposition under humidity stress (Wang Y. H. et al., 2012). By studying changes of Bt insecticidal protein content during the growth duration for different types of Bt cotton varieties, Chen et al. (2003, 2005) found that the activity of GPT and the content of soluble protein were closely positively related to the content of Bt-insecticidal protein, and suggested that overall nitrogen metabolism influenced the expression of Bt-insecticidal protein. Our results also support this model – under soil water deficit, the activities of synthetic enzymes related to nitrogen metabolism (NR and GPT) decreased and the activities of decomposition enzymes (peptidase and protease) increased in boll shells of Sikang1 and Sikang3. The content of insecticidal protein showed significant positive correlation with NR and GPT activities, and significant negative correlation with peptidase and protease activities. Despite higher gene expression, moderately decreased protein synthesis accompanied by a larger increase in protein decomposition resulted in decreased insecticidal protein content of Bt cotton under soil water deficit. This suggests that increased protein decomposition might be the main factor that leads to reduced insecticidal protein, but this hypothesis should be further verified.

In summary, the content of insecticidal protein in boll shells and associated insect resistance decreased when Bt cotton was subjected to soil water deficit stress during bolling, the key period forming yield and yield quality. At a practical level, an important selection criterion is to choose those genotypes which have stronger ability to adapt to soil water deficit stress and use management practices such as timely irrigation, fertilization, and spraying with stress resistance regulators (Xiang et al., 2007; Zhang et al., 2010) to maintain stable and efficient expression of Bt insecticidal protein and provide effective protection of high quality and yield of transgenic Bt cotton.

## Author Contributions

XZ and DC designed the experiments and wrote the manuscript. JW and SP carried out the experiments. YL analyzed the data. XT, GW, and ZZ assisted doing the experiment. ZD and YC helped to draft the manuscript.

## Conflict of Interest Statement

The authors declare that the research was conducted in the absence of any commercial or financial relationships that could be construed as a potential conflict of interest.
